# Puerperal Eclampsia—Its Etiology and Treatment

**Published:** 1897-03

**Authors:** 


### Abstracts and Selections.

### Puerperal Eclampsia—Its Etiology and Treatment.

AUTHOR'S ABSTRACT.

Dr. William Warren Potter, of Buffalo, read a paper at the 91st annual meeting of the Medical Society of the State of New York, Albany, January 26, 1897.

He said, inter alia, that we seem to have arrived at the renaissance of eclamptic literature, that while the subject is being discussed in magazine articles and societies it would not answer for this society to keep silent.

Though the pathogenesis of eclampsia is still unsettled, we are certain that it is a condition *sui generis*, pertaining only to the puerperal state, and that to describe, as formerly, three varieties—hysterical, epileptic and apoplectic—is erroneous as to pathology and causation, as well as misleading in treatment.

The kidney plays an important office in the economy of the eclamptic. If it fails to eliminate toxins, symptoms are promptly presented in the pregnant woman. Renal insufficiency is a usual accompaniment of the eclamptic state. Overproduction of toxins and underelimination by the kidney is a short route to an eclamptic seizure. However, many women with albuminuria escape eclampsia, and many eclamptics fail to exhibit albuminous urine.

The microbic theory of eclampsia has not yet been demonstrated. The toxemic theory in the present state of our knowledge furnishes the best working hypothesis for prevention or cure.

Treatment should be classified into (a) preventive and (b) curative. The preventive treatment should be subdivided into medicinal and hygienic; and the curative into medicinal and obstetric. A qualitative and quantitative analysis of the urine must be made at the onset. If there is defective elimination something must be done speedily to correct a faulty relationship between nutrition and excretion. One of the surest ways to control progressive toxemia is to place the woman upon an exclusive milk diet. This will also serve to flush the kidneys, and thus favor elimination. Distilled water is one of the best diuretics; it increases activity and supplies material—two important elements. In the pre-eclamptic state, when there is a full pulse with tendency to cyanosis, one good full bleeding may be permissible, but its repetition should be regarded with suspicion. If there is high arterial tension—vasomotor spasm—glonoin in full doses is valuable.

When eclampsia is fully established, the first indication is to control the convulsions. Full chloroform anæsthesia may serve a good purpose. If the convulsions are not promptly controlled the uterus must be speedily emptied. This constitutes the most important method of dealing with eclampsia. Two lives are at stake, and by addressing ourselves assiduously to speedy delivery of the fetus we contribute in the largest manner to the conservation of both.

Rapid dilatation first with steel dilators, if need be, then with manual stretching of the os and cervix, followed by the forceps, is the nearest approach to idealism. Only rarely can the deep incision of Duhrssen be required. Cesarean section should be reserved for extreme complications, as deformed pelvis, or to preserve the fetus when the mother's condition is hopeless. Veratrum viride is dangerous, uncertain and deceptive in action.

In eclampsia of pregnancy, i. e., prior to term, the aseptic bougie, introduced to the fundus and coiled within the vagina, may be employed to induce labor. Finally, to promote the elimination of toxic material diuresis, catharsis, and diaphoresis should not be forgotten; neither should the hot air bath, nor the hot pack be overlooked.

